# How multimodal narrative and visual representations of human-like service robots shape attitudes and social connection

**DOI:** 10.3389/frobt.2025.1568146

**Published:** 2025-05-22

**Authors:** Neil Anthony Daruwala

**Affiliations:** School of Psychology, Sport and Health Sciences, University of Portsmouth, Portsmouth, United Kingdom

**Keywords:** human-robot interaction (HRI), multimodal intervention, attitudes toward robots, affective responses, interpersonal connection, human-like service robots, narrative and visual representations

## Abstract

**Introduction:**

Public attitudes toward service robots are critical to their acceptance across various industries. Previous research suggests that human-like features and behaviours perceived as empathetic may reduce negative perceptions and enhance emotional engagement. However, there is limited empirical evidence on how structured multimodal interventions influence these responses.

**Methods:**

A partially mixed experimental design was employed, featuring one between-subjects factor (group: experimental vs. control) and one within-subjects factor (time: pre-intervention vs. post-intervention), applied only to the experimental group. Two hundred twenty-eight adults (aged 18–65) were randomly assigned to either the experimental or control condition. The intervention included images, video demonstrations of human-like service robots performing socially meaningful gestures, and a narrative vignette depicting human–robot interaction. The control group completed the same assessment measures without the intervention. Outcomes included negative attitudes toward robots (Negative Attitudes Toward Robots Scale, NARS), affect (Positive and Negative Affect Schedule, PANAS), and perceived interpersonal connection (Inclusion of Other in the Self scale, IOS).

**Results:**

The experimental group demonstrated a significant reduction in negative attitudes (p < 0.001, Cohen’s d = 0.37), as well as lower negative affect and a greater perceived interpersonal connection with the robots (both p < 0.001). Age moderated baseline attitudes, with younger participants reporting more positive initial views; gender was not a significant factor.

**Discussion:**

These findings suggest that multimodal portrayals of human-like service robots can improve attitudes, affective responses, and interpersonal connection, offering practical insights for robot design, marketing, and public engagement strategies.

## 1 Introduction

As robots are increasingly designed to replicate human behaviours, anthropomorphic designs have become critical in advancing human-robot interaction (HRI). Unlike conventional machines, service robots with humanlike features and behaviours replicate elements of human-to-human dynamics, opening new opportunities for emotional and social connection (McGlone et al., 2014). Despite their rising presence in caregiving, hospitality, and service industries, significant gaps remain in understanding how visual and textual narratives influence attitudes, emotional attachment, and perceptions of closeness factors essential for effective interaction and widespread acceptance of social robots (Joffe, 2008).

Attitudes are fundamental to understanding human behaviour, shaping decision-making, and influencing information processing (Eagly and Chaiken, 1993; Fazio et al., 1995). Attitudes are broadly understood as psychological tendencies that involve evaluating entities with varying levels of favour or disfavour (Eagly and Chaiken, 1993). They guide approach or avoidance motivations. Attitudes are central to fields like social psychology, marketing, and persuasion (Mitchell, 1986; Vogel and Wanke, 2016) as they influence how people perceive and respond to external stimuli. In the human-robot interaction (HRI) context, attitudes are critical in determining robots’ acceptance, rejection, or integration into diverse social and professional settings.

## 2 Attitudes and their role in human-robot interaction

Attitudes toward robots vary widely, ranging from positive to neutral or negative. Positive attitudes are often linked to anthropomorphic robotic features, such as expressive gestures and human-like behaviours, which match users’ emotional expectations (Epley et al., 2007; Eyssel et al., 2012). Eldercare robots designed to evoke trust and companionship have encouraged acceptance among older adults. Neutral attitudes are more situational and influenced by functionality, reliability, and interaction design (Kiraga et al., 2023). Conversely, negative attitudes frequently stem from fears and anxiety (Gnambs and Appel, 2019) or discomfort with overly humanlike designs that may evoke feelings of eeriness or dehumanisation (Akdim et al., 2023).

Recent meta-analytical evidence further supports these variations, indicating that people generally hold favourable attitudes toward social robots and are willing to engage with them. A systematic review by Naneva et al. (2020), synthesising findings from 97 studies (N > 13,000), reported an overall positive valence in public perceptions of social robots, with attitudes moderated by robot design, application domain, type of exposure, and user characteristics. These findings highlight the promise and complexity of social robot acceptance and reinforce the importance of studying how design features and media representations shape emotional responses and trust in human-robot interaction.

### 2.1 Attitude change through interventions

The degree to which attitudes can change is a central question in psychological research. Contemporary theories propose a hybrid model, suggesting that attitudes are partly rooted in memory and partly constructed in response to situational factors (Albarracín, 2021; Maio et al., 2019). To shape public attitudes positively, leveraging videos and imagery has proven effective. Visual media portraying robots in positive roles can reduce fear and increase perceptions of competence and warmth (Orefice et al., 2016). Videos demonstrating robots’ capabilities in healthcare or hospitality familiarise audiences with their functionality and evoke emotional engagement, encouraging more favourable attitudes (Broadbent et al., 2009).

These visual strategies are rooted in dual-process models like the Elaboration Likelihood Model (ELM) (Briñol and Petty, 2011). By leveraging the peripheral route of persuasion, engaging videos and imagery can create positive effects and influence attitudes without requiring extensive cognitive processing (Briñol and Petty, 2011). Such interventions promote public acceptance and could promote collaboration in HRI contexts.

Building on these insights, this study explores how early-stage visual and textual representations of robots can positively shape attitudes in the service industries (Stafford et al., 2014). Their work highlighted that preexisting mental models significantly influence how participants evaluate robots, often outweighing factors like human or machine likeness or even the presence of a virtual face. Their study employed visual methods like participant-generated robot drawings to measure attitude shifts pre- and post-intervention.

Expanding on this work, the present study investigates how targeted human like cues specifically empathetic and positive gestures, imagery, and textual narratives can reduce negative perceptions and enhance perceived interpersonal connection and affect toward service robots towards robots. By employing carefully designed visual and textual interventions, this research aims to demonstrate how public attitudes can be positively influenced, facilitating broader acceptance of robots across social and professional contexts. This approach is further supported by Leite et al. (2013), which showed that when a robot displayed empathetic behaviours such as expressive facial cues and verbal reinforcement, it was perceived as significantly friendlier and more emotionally engaging. These findings validate the hypothesis that empathetic cues, including positive gestures like handshakes, are vital in shaping effective human-robot relationships and fostering trust and social connection.

### 2.2 Attitudes towards anthropomorphic robots

Anthropomorphic robots, characterised by their human like design and capacity for social interaction, represent a convergence of technological innovation and human-centric design principles. According to the Media Equation Theory (Reeves and Nass, 1996), humans interact with these robots as social agents, perceiving them as more than mere tools. This perception is reinforced by positive robot expressions, which enhance user acceptance and satisfaction. However, balancing novelty and relatability is crucial, as overly familiar designs can reduce user enjoyment (Bishop et al., 2019). Humanlike appearances often generate favourable attitudes by evoking familiarity and compatibility with established human mental models (Epley et al., 2007; Pillai and Sivathanu, 2020; Qian and Wan, 2024). Restaurant patrons exhibited higher levels of engagement with anthropomorphic robots that displayed moderate human likeness, with attitudes further improving when a sense of rapport was established during interactions (Qiu et al., 2020).

Nonetheless, the results surrounding attitudes towards human like service robots remain varied and challenging to generalise. While the uncanny valley hypothesis posits that robots with high levels of human resemblance may provoke discomfort or even aversion, other findings suggest that low to moderate humanlike features evoke the most positive responses by balancing familiarity and distinctiveness (Gnambs and Appel, 2019; Akdim et al., 2023; Mara et al., 2022). In frontline service roles, highly anthropomorphic robots can sometimes be perceived as impersonal or unsettling due to their perceived lack of authenticity. In contrast, less humanlike robots are often preferred for their simplicity and functionality, particularly in automated environments (Kazandzhieva and Filipova, 2019; Yu, 2020). These contrasting outcomes underscore the complexity of designing practical anthropomorphic robots that users positively receive.

This study employs a multimodal intervention using textual narratives and imagery, including compositions and video demonstrations, to examine potential changes in attitudes toward human like service robots, as measured by the Negative Attitudes Toward Robots Scale (NARS; Nomura et al., 2006). Perceived interpersonal closeness with the robots is assessed using the Inclusion of Other in the Self scale (IOS; Aron et al., 1992), and emotional affect is measured using the Positive and Negative Affect Schedule (PANAS; Watson et al., 1988). By integrating these narrative and visual elements, the study seeks new insights into how human perceptions and emotional responses evolve in varying contexts, providing actionable evidence to inform future design and implementation strategies.

## 3 Visual and textual cues in HRI and marketing applications

Visual and textual cues are critical in shaping attitudes toward anthropomorphic robots by activating pre-existing associations and eliciting emotional responses. The Associative-Propositional Evaluation (APE) model (Gawronski and Bodenhausen, 2006) offers a valuable theoretical framework for understanding how such cues activate associative processes, leading to automatic positive evaluations. Vivid visual representations, including concrete images and narratives, enhance mental visualisation and strengthen favourable attitudes (Babin and Burns, 1997). Demonstrated that visual imagery mediates attitudes and improves perceptions of advertisements and brands (Dasgupta and Greenwald, 2001). Showed that positive visual exemplars can shift implicit attitudes, even when explicit evaluations remain unchanged. These findings highlight the potential for visual and textual cues to create emotional connections and improve perceptions of anthropomorphic robots.

In industries such as hospitality and tourism, where emotional engagement is central, anthropomorphic traits significantly influence consumer acceptance of robotic services (Murphy et al., 2021). The researchers argue that marketing strategies in these sectors should emphasise humanlike features to enhance the appeal of service robots.

Consumer perceptions were analysed using video examples from the Hennna Hotel in Japan, the world’s first robot-operated hotel. The study demonstrated how anthropomorphic imagery increases user attitudes and acceptance (Yu, 2020). Furthermore, humanlike gestures and expressions in robots promote familiarity through shared cognitive schemas (Epley et al., 2007) and increase perceptions of warmth, trust, and animacy (Orefice et al., 2016). These characteristics make robotic service providers more relatable, particularly in emotionally driven industries.

Despite these advancements, the mechanisms through which visual and textual cues influence attitudes and emotional engagement in human-robot interaction (HRI) remain underexplored. While (Qiu et al., 2020) emphasises the importance of mediated visual cues, such as simulated affective touch, in enhancing emotional connections and (Iwamoto et al., 2022) highlights how multimodal interventions using narrative and visual representations can strengthen affective responses, there is limited research investigating how these cues operate across marketing and HRI contexts. To address this gap, the present study integrates vivid visual and textual representations to examine how humanlike service robots can be effectively portrayed to improve consumer perceptions. This dual focus on HRI and marketing has practical implications for designing advertising and promotional strategies, particularly in hospitality, caregiving, and tourism, where emotional engagement with robotic technology is essential. Complementing this (Suzuki et al., 2015), demonstrated that emotional reactions to visual robot stimuli can be elicited at a physiological level, showing empathic neural responses through electroencephalographic measures. Their use of ANOVA-based analysis of affective states aligns with the present study’s methodological approach and reinforces the relevance of visual stimuli in shaping emotional engagement with robots.

### 3.1 Visual and textual interventions

Visual and textual cues are powerful for shaping user perceptions, particularly when direct interaction is impractical. Videos depicting robots performing gestures, such as handshakes, offer controlled scenarios for evaluating emotional responses by enabling researchers to manipulate variables like social context and robot behaviour (Cianchetti et al., 2014). Gestures like handshakes evoke empathy, trust, and warmth, even without physical interaction (Nakanishi et al., 2014; Rosenthal-von der Pütten et al., 2013). Similarly, realistic visual representations enhance a sense of presence and engagement (Shen et al., 2011), while narratives describing empathetic behaviours increase perceptions of trustworthiness and animacy (Law et al., 2021; Orefice et al., 2016; Willemse and van Erp, 2018).

Embodied cognition theory suggests that human thought is not abstract but shaped by bodily experiences and interactions with the physical world (Barsalou, 2008; Wilson, 2002; Glenberg, 2010). Within this framework, exposure to multimodal stimuli, such as visual media and narratives, can activate perceptual and affective simulations, contributing to emotional engagement and attitude formation. Building on these principles, the current study employs videos, imagery, and narrative vignettes to evoke embodied responses that may influence perceptions of anthropomorphic service robots. A video of a human-like robot shaking hands with a customer might evoke positive emotional responses, enhance attitudes toward the robot, and strengthen the perception of interpersonal connection between the user and the service robot (Gallese and Ebisch, 2016). Prior research demonstrates that mediated social touch activates neural regions associated with physical touch, creating a sense of connection without direct interaction (Erk et al., 2015). Inspired by Law et al. (2021), this study includes scripted vignettes to assess attitudes and interactions with robotic technologies, as narratives are practical tools for eliciting responses and insights.

By integrating visual and textual interventions, this research aims to shift attitudes and emotional responses by engaging participants’ imagination and empathy. These findings will inform scalable and cost-effective approaches for enhancing human-robot interaction across the service industries. Based on this foundation, the study hypothesises:

• H1a: Participants in the experimental group will exhibit a significant change in attitudes toward service robots, as evidenced by differences in Negative Attitudes Toward Robots Scale (NARS) scores before and after the intervention.
• H1b: Participants exposed to visual and textual interventions will report a higher positive affect on service robots, demonstrating the interventions’ impact on shaping perceptions.
• H1c: It is hypothesised that participants exposed to the multimodal intervention will demonstrate significantly greater perceived interpersonal connection with service robots, as assessed by the IOS scale, compared to those in the control group.

## 4 Gender differences

Emotional engagement, defined as users’ ability to form meaningful connections with robots, is critical in shaping positive attitudes and perceptions of closeness. Empathetic cues and contextual narratives may resonate more effectively with women, while men may respond more positively to designs emphasising functionality and innovation. Research indicates that women and men prioritise different aspects of their robot interactions. Women often value trust and empathy more, strengthening emotional connections (Andreasson et al., 2018). Men, by contrast, tend to focus on technological novelty and utility, adopting a more functional perspective (Lee and Yen, 2023). These differences highlight the importance of gender-sensitive service robot portrayals, where empathy is essential, compared to innovation-driven settings, where men may exhibit more positive attitudes (Kuo et al., 2009). Utilising the same scale applied in this study (Nomura and Suzuki, 2022), found that gender stereotypes and negative attitudes toward robots (NARS) influenced preferences for robot appearance; however, the extent of this impact varied depending on the specific human occupation.

• H2a: Women will exhibit higher baseline negative attitudes toward humanlike robots than men (Szczuka and Krämer, 2018).
• H2b: Women will demonstrate higher emotional engagement with humanlike robots than men

## 5 Age differences

Age is another critical factor influencing attitudes toward robots, particularly concerning emotional engagement and physical interaction. Older adults often express discomfort with emerging technologies due to limited familiarity or confidence (Kuo et al., 2009; Daruwala, 2024). However, humanlike robots can mitigate these challenges where emotional connection is a priority (Feingold Polak et al., 2018). Older adults experiencing loneliness highly value tactile interactions with robots, which provide comfort and companionship.

• H3a: Older participants will hold more negative attitudes toward service robots.
• H3b: It is hypothesised that, following the intervention, older participants in the experimental group will show significantly higher levels of perceived interpersonal connection (IOS) and positive affect (PANAS) than older participants in the control group.

Interestingly, older adults who initially hold negative views of humanlike robots may become more open to interaction when exposed to positive visual and textual imagery. Videos, narratives, and other interventions depicting robots as empathetic and approachable have the potential to reshape elderly perceptions. This study aims to demonstrate that such interventions can improve emotional engagement and serve as practical tools for promoting service robots as trusted companions.

## 6 Methods

### 6.1 Participants and sampling

Initially, 236 individuals began the survey. After applying exclusion criteria, including incomplete responses (n = 6) and failure to meet eligibility requirements (n = 2). A final sample of 228 participants was retained for analysis. (mean age = 35.30, SD = 11.45; 64% women, 36% men) were recruited through online platforms, including social media and robotics forums, using convenience sampling. The age distribution was as follows: 45% aged 18–30, 35% aged 31–45, and 20% aged 46–65. *A priori* sample size calculations conducted using G*Power (Faul et al., 2007) ensured sufficient statistical power (0.80) to detect medium effect sizes (d = 0.5) at α = 0.05. Participants with significant prior experience with robots were excluded to minimise familiarity bias. Baseline demographic data, including age, gender, and prior exposure to robots, were collected to account for individual differences during analysis. Participation in this study did not offer direct financial or material benefits; however, participants were informed that their responses would contribute to advancing academic knowledge in human-robot interaction and service technology design.

### 6.2 Experimental design and intervention

This study employed a partially mixed experimental design, incorporating one between-subjects factor (group: experimental vs. control) and one within-subjects factor (time: pre-intervention vs. post-intervention), applied only within the experimental group. Participants were randomly assigned via a Qualtrics randomiser block to one of two conditions: an Experimental Group (who received the intervention materials) or a Control Group (who received no intervention materials). Each condition followed a different assessment pathway:

Experimental Group (n = 114): Exposed to visual and textual interventions simulating human-robot interactions.

Control Group (n = 114): Completed assessments without exposure to intervention materials.

Still Image from Intervention Video Depicting Affective Touch Gesture During Human–Robot Handshake Interaction (EZ MODE, 2024).

### 6.3 Intervention materials

**FIGURE 1:**
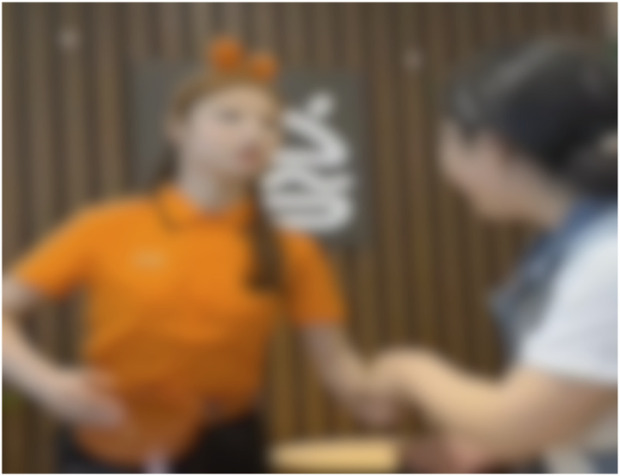
Note. This image was used in the experimental group intervention to simulate affective touch and promote emotional engagement through a humanlike robot performing a socially warm gesture (handshake). The visual was designed to evoke familiarity and increase perceived interpersonal connection with the robot.

**FIGURE 2:**
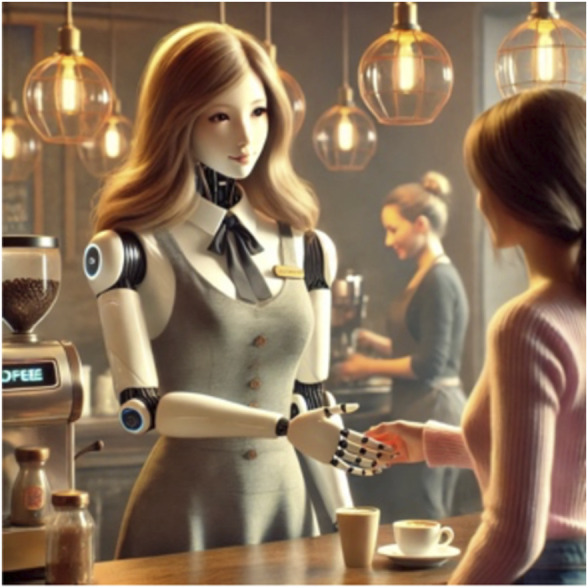
AI-Generated Depiction of Affective Touch Gesture in Human-Robot Interaction within a Service Context. Note. The author created this image using Image Generator Pro (Pulsr, 2024). The depiction was designed to elicit emotional engagement and enhance perceived interpersonal connection through the portrayal of a socially meaningful touch gesture.

The intervention materials were grounded in embodied cognition theory and designed to evoke affective responses and perceived interpersonal connection with humanlike service robots. These materials included: Video Presentation: A 30-s video of a humanlike robot shaking hands with a customer in a simulated café setting, emphasising warmth and naturalistic gestures (Figure 1). Images: High-resolution photographs depicting soft robots engaging in humanlike interactions, such as serving customers (Figure 2). Vignette/Story Script: A narrative describing a humanlike robot waitress interacting with a customer, designed to evoke emotional engagement and enhance perceptions of interpersonal connection between the human and the robot (see Appendix A). The intervention materials were standardised in a 10–15-min sequence (video, images, and vignette) and pilot-tested with a small sample (n = 10) to confirm their effectiveness in eliciting attitudinal and affective responses, and perceived interpersonal connection.

The image (Figure 1) is taken from a royalty-free video on YouTube (EZ MODE, 2024); however, it is blurred as a precaution due to possible copyright infringements. The image (Figure 2) was explicitly designed using Pulsr AI’s Image Generator Pro (Pulsr, 2024) to depict an humanlike service robot waitress shaking hands with a human client. The visual aims to evoke emotional affect and tactile engagement as part of the intervention viewed by the experimental group. This image was a controlled stimulus for assessing participants’ attitudes toward robot-human interactions. The Acknowledgements section at the end of this paper provides further details about generative AI tools, including their acknowledgement.

### 6.4 Assessment procedure and measurement instruments

**FIGURE 3:**
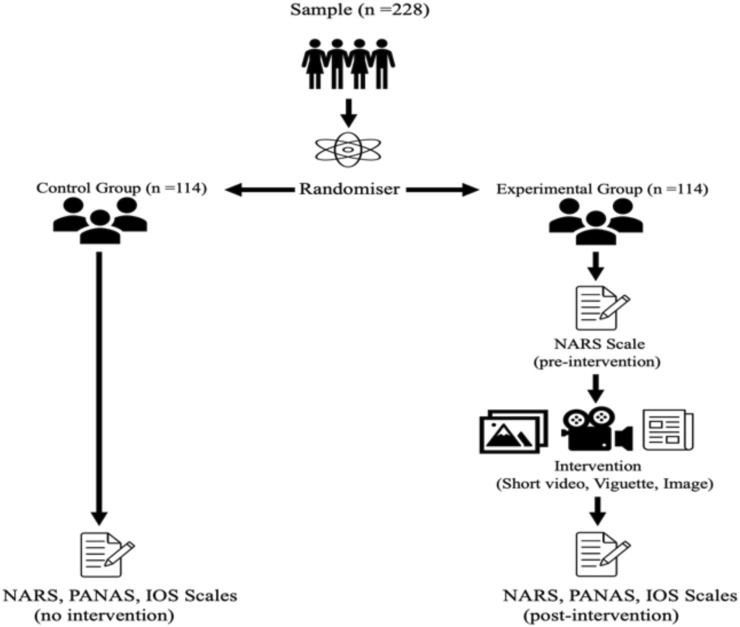
A schematic overview of participant randomisation and assessment timing across intervention and control conditions.

Data were collected across three conditions: pre-intervention, post-intervention, and no-intervention (control group), to evaluate changes in participant attitudes, emotional responses, and perceived interpersonal closeness toward service robots (See Figure 3).

### 6.5 Pre-intervention (baseline for both groups)

All participants completed the following assessments prior to any intervention:

Negative Attitudes Toward Robots Scale (NARS):

A 14-item validated measure assessing participants’ baseline attitudes toward robots. It includes three subscales: Situational Interaction (SIT): α = 0.67, Emotional Interaction Response (EIR): α = 0.75, Societal Influence (SCT): α = 0.81.

Demographic Survey:

Information on age, gender, and previous exposure to robots was collected to control for individual variation.

### 6.6 Post-intervention (experimental group only)

Participants assigned to the experimental group were exposed to a multimodal intervention consisting of:

• A short video
• A narrative vignette
• Still imagery of a service robot performing prosocial tasks.

Following this exposure, they completed:

• NARS (Post-Intervention): To measure any change in attitudes toward robots.
• Positive and Negative Affect Schedule (PANAS): To assess participants’ emotional states.
• Inclusion of the Other in the Self (IOS) Scale: This single-item pictorial measure was used to assess perceived interpersonal connection, reflecting the extent to which participants felt relational overlap or closeness with the robot (Aron et al., 1992).

No-Intervention (Control Group):

Participants in the control group did not receive any visual or narrative materials. However, to maintain comparability between groups, they completed the same PANAS and IOS scales following the pre-intervention phase. Notably, both assessments were accompanied by brief scenario-based prompts designed to contextualise responses:

• Prior to completing the PANAS, participants were presented with the following instructions:

Please indicate to what extent each of the following emotional responses would describe how you would feel in the following situation: If a human-like service robot offers you a handshake as part of a socially engaging greeting protocol.

• Similarly, prior to the IOS task, participants were instructed:

Please indicate your perceived interpersonal connection toward human-like service robots by selecting the image that best represents your relationship. The images show varying degrees of overlap between ‘You’ and ‘X’ (the robot). Greater overlap reflects greater perceived closeness or emotional connection.

These contextual prompts ensured that even control group participants engaged with the concept of human-robot interaction, providing a meaningful benchmark against which the experimental group’s emotional and closeness outcomes could be compared.

### 6.7 Instruments

Negative Attitudes Toward Robots Scale (NARS):

This is a 14-item measure assessing attitudes toward robots, with subscale reliabilities of interaction (α = 0.67), social influence (α = 0.81), and emotional response (α = 0.75) (Nomura et al., 2006).

Positive and Negative Affect Schedule (PANAS) Short Form: Measured emotional responses on a five-point scale (e.g., inspired, upset) (Thompson, 2007; Watson et al., 1988).

**FIGURE 4:**
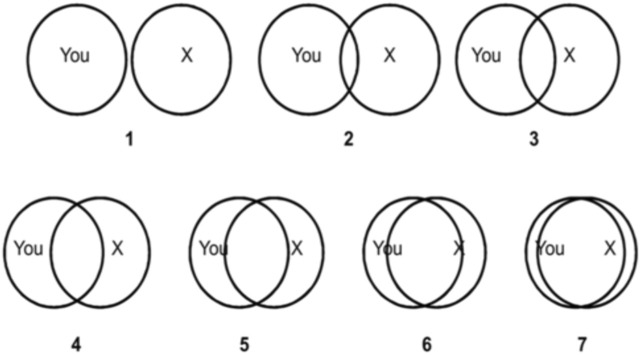
The ‘Inclusion of the Other in the Self’ (IOS). *Note.* Inclusion of Other in Self (IOS) Scale X represents perceived closeness with soft robotic haptic technology.

Inclusion of the Other in the Self (IOS): Assessed perceived interpersonal connection/closeness using overlapping circle diagrams (Aron et al., 1992) (Figure 4).

### 6.8 Procedure

Participants first completed baseline assessments using the NARS to establish initial attitudes toward robots. The experimental group was then exposed to the intervention materials, while the control group received no intervention. Both groups completed post-intervention measures of PANAS and IOS to evaluate emotional responses and perceived closeness. A subset of experimental group participants (n = 30) completed the NARS at a follow-up point to evaluate the stability of attitude changes over time.

### 6.9 Data analysis

Data were analysed using SPSS 28.0.1.1, employing the following methods: Descriptive Statistics and Correlations: Explored relationships among demographic variables and outcomes. Paired Samples t-tests: Assessed pre- and post-intervention differences in NARS scores for the experimental group. Two-Way ANOVA: Compared PANAS and IOS scores between the experimental and control groups. Ethical Considerations: Participants were informed about the study’s goals and procedures and provided written informed consent before participation. The corresponding university ethics committee granted ethical approval. Participants were assured of data anonymity and debriefed upon completing the study.

## 7 Results

All statistical analyses presented in this section are based on the final sample (N = 228), following the exclusion of participants due to incomplete responses (n = 6) and eligibility non-compliance (n = 2).

### 7.1 Descriptive Statistics

Descriptive statistics were computed to summarise participants’ demographic characteristics and baseline attitudes toward robots (See Table 1), as measured by the Negative Attitudes Toward Robots Scale (NARS). The sample consisted of 228 participants aged 18–65 (mean age = 35.30, SD = 11.45), balanced by gender (50% male, 50% female).

**TABLE 1:** Descriptive statistics (means and standard deviation for gender).

Age group	Gender	Mean negative Attitude to robots	Standard deviation	n
18-30	Male	3.05	0.35	40
	Female	2.95	0.28	40
31-45	Male	3.12	0.34	45
	Female	3.08	0.30	45
46-65	Male	3.15	0.36	37
	Female	3.12	0.31	37

Note. standard deviations are shown in parentheses.

Baseline NARS scores indicated moderate negative attitudes toward robots (M = 3.10, SD = 0.59).

### 7.2 H1a: Impact of Interventions on attitudes toward robots (NARS)

A paired samples t-test was conducted to assess changes in attitudes toward service robots using the NARS scale in the experimental group. Participants reported significantly lower negative attitudes post-intervention (M = 2.89, SD = 0.51) compared to pre-intervention scores (M = 3.10, SD = 0.59), t (113) = 60.46, p < 0.001, 95% CI [0.18, 0.24]. These results support H1a, indicating that the visual and textual interventions significantly reduced negative attitudes toward service robots.

Interpretation: This supports H1a, confirming that visual and textual interventions significantly improved attitudes toward service robots.

### 7.3 H1b: Effect of interventions on positive affect (PANAS)

**FIGURE 5:**
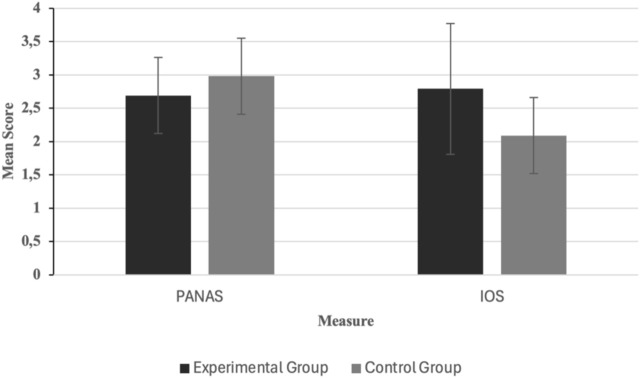
Mean scores for affective responses (PANAS) and perceived interpersonal connection (IOS) across experimental and control groups. Error bars represent standard deviations.

A two-way ANOVA revealed that participants in the experimental group reported significantly lower negative affect (M = 2.69, SD = 0.57) than those in the control group (M = 2.98, SD = 0.57), F (1,113) = 55.91, p < 0.001. This supports H1b, demonstrating the interventions’ positive influence on emotional states (See Figure 5).

### 7.4 Perceived interpersonal connection post-intervention (IOS)

Analysis revealed a significant group difference in IOS scores, indicating that participants in the experimental group experienced greater perceived interpersonal connection with human-like service robots compared to those in the control group (M = 2.79, SD = 1.01) than the control group (M = 2.09, SD = 0.57), F (1,113) = 29.50, p < 0.001. H1c is therefore supported, confirming the role of narrative and imagery in enhancing affective proximity to human like service robots.

### 7.5 Hypothesis H2a gender differences in negative attitudes (baseline NARS)

Independent samples t-tests assessed gender differences in baseline NARS scores.

• Women: M = 3.04, SD = 0.32
• Men: M = 3.09, SD = 0.39
• Result: t (226) = 1.12, p = 0.27
• Interpretation: No significant gender difference was found. H2a is not supported.

### 7.6 Hypothesis H2b–Gender differences in emotional engagement

Pearson correlation analysis revealed a positive association between gender and negative affect (r = 0.138, p = 0.038), suggesting that female participants may have reported higher levels of negative emotional responses. However, further analysis is recommended to examine potential gender differences in perceived interpersonal connection and affective responses to ensure more robust interpretation of these findings.

### 7.7 Hypothesis H3a–Age differences in attitudes

**FIGURE 6:**
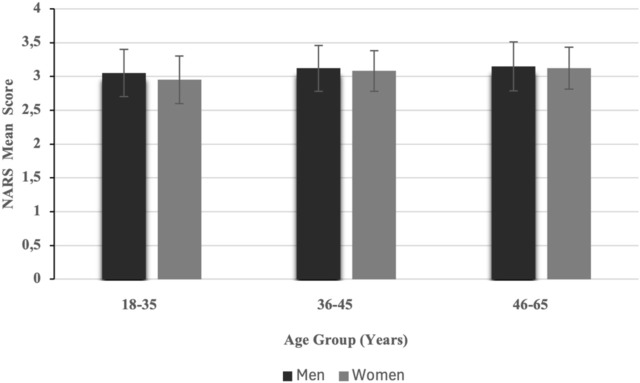
Mean negative attitudes toward robots (NARS) scores by gender and age group.

A Pearson correlation analysis showed a small but significant negative relationship between age and NARS scores (r = −0.197, p = 0.003), indicating older participants held more negative attitudes toward robots (See Table 2). This supports H3a (See Figure 6).

**TABLE 2:** *Pearson Correlations Between Age, Gender, Affect, Closeness, and Negative Attitudes Toward Robots*.

	Gender	Age	NEGA	PA	PANAS	IOS	NARS
Gender	1						
Age	0.108 (0.105)	1					
NA	0.138* (0.038)	−0.092 (0.167)	1				
(PA)	−0.129 (0.052)	−0.140* (0.035)	0.069 (0.300)	1			
PANAS	0.020 (0.764)	−0.155* (0.019)	0.778** (<0.001)	0.681** (<0.001)	1		
IOS	−0.056 (0.401)	−0.202** (0.002)	−0.337** (<0.001)	0.334** (<0.001)	−0.038 (0.565)	1	
NARS	−0.058 (0.381)	−0.197** (0.003)	0.374** (<0.001)	0.145* (0.029)	0.366** (<0.001)	0.074 (0.267)	1

Note: *Correlation is significant at the 0.05 level (2-tailed). **Correlation is significant at the 0.01 level (2-tailed).

### 7.8 Hypothesis H3b–Age-based emotional responses post-intervention

Correlational analysis revealed that age negatively correlated with:

• Positive Affect (r = −0.140, p = 0.035),
• PANAS (r = −0.155, p = 0.019),
• IOS (r = −0.202, p = 0.002)

Although older participants demonstrated lower levels of positive affect post-intervention, the pattern indicates modest improvement, particularly in affective responses and perceived interpersonal connection with the service robots. Partial support for H3b is observed, though more robust group-based analyses are encouraged.

Results strongly support primary hypotheses related to intervention effectiveness (H1a–c). While gender-related hypotheses (H2) were only partially supported, age-related attitudes (H3) were validated through correlation analysis. Collectively, findings emphasise the value of visual/textual emotional engagement tools in shaping public perceptions of robots.

## 8 Discussion

### 8.1 Overview

This study investigated the effects of visual and textual interventions on emotional affect, perceived closeness, and attitudes toward human like service robots. The findings demonstrate that the intervention significantly increased perceived interpersonal connection and positive affect, while reducing negative attitudes toward humanlike service robots, through the use of crossmodal sensory cues designed to simulate humanlike interactions. Participants in the experimental group exhibited lower negative attitudes (NARS), higher perceived interpersonal connection (IOS) and positive affect (PA) than the control group, illustrating early-stage interventions’ potential to shape user perceptions positively (see Table 3). These results provide scalable methods to influence public attitudes and promote broader acceptance of robotic technologies where humanlike robots will be increasingly utilised.

**TABLE 3:** Pre- and post-intervention changes in attitudes, affect, and closeness scores (NARS, PANAS, IOS).

Measure	Mean	Std. Deviation	Std. Error mean	95% CI lower	95% CI Upper	t	df	p
Pre-intervention (NARS)	3.10	0.59	0.07	2.98	3.22	-	-	-
Post-intervention (NARS)	2.89	0.51	0.036	2.82	2.96	60.46	113	<0.001
Post-intervention (PANAS)	2.69	0.57	0.06	2.57	2.81	-	-	-
Difference (NARS pre – PANAS post)	0.41	0.59	0.07	0.28	0.54	6.21	81	<0.001
PANAS CONTROL	4.57	1.00	0.09	4.38	4.76	48.83	113	<0.001
Post-intervention (IOS)	2.09	0.57	0.06	1.97	2.21	-	-	-
Difference (NARS - IOS)	1.01	1.28	0.09	0.84	1.18	11.83	225	<0.001
IOS CONTROL	4.52	1.10	0.10	4.31	4.73	43.88	113	<0.001

Note. CI, Confidence Interval. the table presents the means, standard deviations, standard errors, confidence intervals, t-values, degrees of freedom (df), and p-values for the paired samples t-test comparing overall negative attitudes towards soft robots before and after watching a video. The effect sizes are reported using Cohen’s d and Hedges’ g, with corresponding confidence intervals.

Additionally, positive visual and textual imagery offers valuable applications for marketing campaigns. Enhancing attitudinal and emotional responses presents an accessible, cost-effective approach to improving trust and emotional connection with robotic technologies. This investigation highlights the importance of visual and textual cues in creating meaningful human-robot interactions and demonstrates how targeted strategies can shift perceptions in diverse contexts.

### 8.2 Perceptions of robotic touch through multimodal narrative and visual interventions

This study demonstrated that visual and textual interventions significantly influenced participants’ attitudes (H1a), emotional affect (H1b), and perceived interpersonal connection (H1c) toward humanlike service robots. Quantitative analyses revealed a reduction in negative attitudes toward robots (NARS) post-intervention, with a medium effect size (d = 0.41). Participants in the experimental group also reported significantly lower negative affect (PANAS) and increased positive affect, with a medium effect size (d = 0.51). Finally, perceived interpersonal connection (IOS) increased substantially, with a large effect size (d = 0.84). These findings suggest that sensory-based interventions, such as videos and vignettes, improve emotional engagement and user perceptions of service robots.

### 8.3 Interpretation of results

**FIGURE 7:**
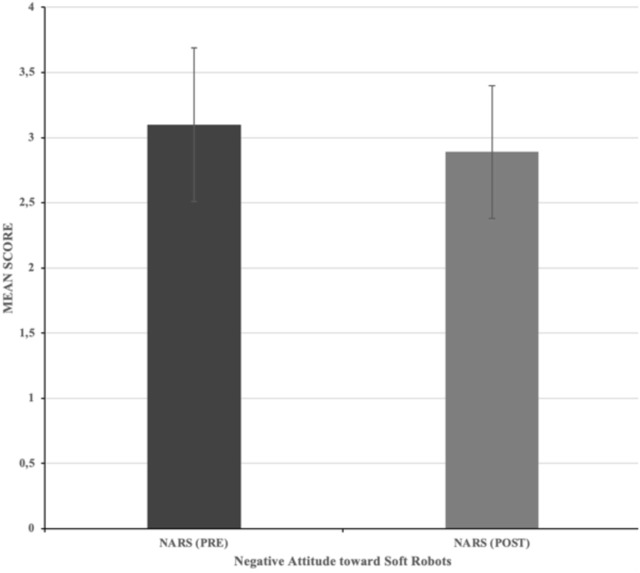
Impact of Intervention on NARS Pre- and Post-Intervention Scores. Note. Error bars represent the standard deviation.

The reduction in negative attitudes (H1a) is consistent with prior research showing that humanlike cues, such as expressive gestures and empathetic behaviours, positively influence perceptions of robots (Eyssel et al., 2012; Willemse and van Erp, 2018). By employing multimodal interventions (visual and textual), this study demonstrates that attitudes can be improved (See Figure 7) before direct interaction occurs, addressing the influence of pre-existing mental models on robot acceptance (Stafford et al., 2014). Furthermore, the increased emotional affect (H1b) supports embodied cognition theory, which proposes that imagined sensory experiences evoke emotional responses comparable to physical interactions (Barsalou, 2008).

Crucially, the results contrast with those of Wullenkord and Eyssel (2014), who found that imagined contact with robots did not improve attitudes. This discrepancy likely stems from methodological and contextual differences: Imagined contact lacks the vividness of visual-textual stimuli, which may no longer be sufficient in today’s more robot-saturated cultural landscape. In contrast, the study’s direct and immersive interventions engaged participants more effectively, reflecting current societal familiarity with robotic technologies in media and daily life.

Notably, the findings challenge the uncanny valley hypothesis. While this hypothesis suggests that robots with highly humanlike features may provoke discomfort or aversion (Gnambs and Appel, 2019; Akdim et al., 2023), this study demonstrates that humanlike service robots can elicit positive emotional responses when introduced through relatable imagery and narratives. Participants in the experimental group rated the service robot portrayed in videos and vignette as warmer, more likeable, and emotionally engaging than the control group. These results support previous research showing empathy and relatable traits improve trust and connection with robots (Eyssel et al., 2012; Nakanishi et al., 2014; Rosenthal-von der Pütten et al., 2013; Shen et al., 2011; Law et al., 2021).

These findings are consistent with dual-process models, such as the Elaboration Likelihood Model (Briñol and Petty, 2011), which explain how peripheral cues, like engaging visuals and narratives, influence attitudes without extensive cognitive processing. Sensory simulations, such as visuotactile imagery, further activate neural regions associated with physical touch, creating a sense of connection without direct interaction (Erk et al., 2015; Seinfeld et al., 2022).

### 8.4 Implications and significance

The findings demonstrate that visual and textual interventions can effectively influence user perceptions by improving attitudes, affective responses, and interpersonal connection with human-like service robots. Relatable scenarios and gestures, such as handshakes, have been shown to humanise robots, making them more approachable and trustworthy. This approach is particularly relevant in the caregiving, hospitality, and service industries, where emotional engagement is closely associated with trust, warmth, and user satisfaction (Epley et al., 2007; Willemse and van Erp, 2018).

Although attitudes toward humanlike service robots vary widely, ranging from scepticism to enthusiasm (Naneva et al., 2020), this study demonstrates that carefully designed interventions can reduce hesitations and promote positive perceptions. Such strategies encourage acceptance by bridging the gap between novelty and familiarity, especially in roles requiring an emotional connection.

## 9 Baseline negative attitudes toward robots

The findings revealed that women reported slightly lower baseline negative attitudes toward robots (*M* = 3.04, *SD* = 0.32) compared to men (*M* = 3.09, *SD* = 0.39), though the difference was not statistically significant. This suggests that both genders expressed similar levels of scepticism before the intervention, consistent with previous research highlighting the influence of pre-existing mental models on how robots are evaluated during and after interactions (Andreasson et al., 2018). These baseline attitudes may reflect broader societal and cultural narratives about automation and technology, often presenting robots as helpful innovations or potential threats to employment and social stability.

Interestingly, (Nomura and Suzuki, 2022), findings suggest that perceptions of robots may vary depending on the participant’s occupation. Their study demonstrated that individuals working in innovation-driven fields, such as technology or engineering, exhibited more favourable attitudes toward robots than those in caregiving or emotionally driven professions, who expressed higher scepticism. This highlights the importance of understanding how occupational contexts shape user expectations and perceptions of robots. Designing robots for specific roles may benefit from consideration of these occupational differences, particularly in empathy-oriented industries where emotional engagement is critical.

The results also support (Lee and Yen, 2023) argument that preexisting mental models of robots can be more influential than specific robot features, such as humanlike characteristics or virtual facial expressions, in shaping user perceptions. Strategies such as videos or narratives that present service robots as relatable and beneficial may provide a cost-effective way to improve acceptance. These findings indicate that portraying robots positively before interaction could be just as important, if not more so, than tailoring robots to individual user preferences.

## 10 Comparison with implicit measures and physiological responses

While this study utilised self-reported measures such as NARS, implicit measures could offer additional insights into underlying attitudes and anxieties. Studies have found that participants’ drawings of robots often reveal subconscious fears or expectations that standard surveys may not capture. Research by (Kuo et al., 2009) demonstrated that larger robot drawings were associated with increased physiological responses, such as heightened blood pressure, potentially indicating discomfort or anxiety during interactions. Incorporating such measures in future studies could provide a more comprehensive understanding of user perceptions, particularly when exploring differences across demographics or occupational groups.

These findings highlight the complexity of designing robots that appeal to a wide range of users. Occupational differences, gender-sensitive portrayals, and interventions that depict robots in empathetic and relatable roles may prove especially effective in reducing apprehension and encouraging acceptance. This approach is fundamental in service-oriented industries and emotionally driven fields, such as healthcare and education, where robots must be perceived as functional tools and socially and emotionally engaging entities.

## 11 Emotional engagement post-intervention

The study revealed that women in the experimental group exhibited significantly higher emotional engagement post-intervention, as evidenced by their increased perceived interpersonal connection scores (M = 2.79, SD = 1.01). These findings are consistent with previous research indicating that humanlike gestures and contextual narratives enhance perceptions of warmth, empathy, and trust in robots (Epley et al., 2007; Orefice et al., 2016). The results suggest that women place greater importance on interpersonal connections and relational dynamics in human-robot interactions, a trend noted in prior studies by Andreasson et al. (2018) and Lee and Yen (2023). Such qualities are particularly critical in emotionally driven roles, where social dynamics are pivotal in user acceptance.

The interventions utilised familiar social behaviours, such as a handshake, alongside engaging narratives to improve perceptions of robots and enhance their relatability. Gestures like handshakes evoke positive emotions, including trust and empathy, even without direct physical interaction (Nakanishi et al., 2014; Rosenthal-von der Pütten et al., 2013). These findings support embodied cognition theory, which posits that mediated sensory cues, such as videos and scripted vignettes, activate shared cognitive schemas and generate positive emotional responses (Barsalou, 2008). Additionally, realistic visual representations employed in the interventions further strengthened emotional connections and heightened participants’ sense of engagement with studies (Shen et al., 2011; Gallese and Ebisch, 2016).

Notably, the interventions were effective across all participants, irrespective of gender. The study observed a significant reduction in negative attitudes (M = 2.89, SD = 0.51; t (113) = 60.46, p < 0.001) and negative affect (M = 2.69, SD = 0.57), demonstrating that empathetic gestures and familiar narratives improve emotional engagement and diminish reservations across diverse user groups. These results echo findings by (Qian and Wan, 2024; Iwamoto et al., 2022; Law et al., 2021), which emphasised the role of visual and textual cues in enhancing emotional connections in human-robot interaction (HRI) and marketing contexts.

## 12 Age-related trends in negative attitudes

Baseline measurements revealed that older participants had significantly higher negative attitudes toward robots, as measured by the Negative Attitudes Toward Robots Scale (NARS). A negative correlation between age and NARS scores (*r*−0.197, *p*0.003) confirmed that younger adults were generally more accepting of robots and had lower scepticism toward emerging technologies.

Despite these baseline differences, the study’s interventions effectively reduced negative attitudes across all age groups. Paired samples t-tests revealed a significant reduction in NARS scores post-intervention (*M*2.89, *S D*0.51; *t* ( 113)60.46, *p* < 0.001). Visual and textual cues, such as handshake gestures and empathetic narrative framing, successfully humanised robots and enhanced their relatability.

The results support previous studies that emphasise the role of age in shaping attitudes toward technology. Lee and Yen (2023) found that older adults often approach robots with scepticism due to their unfamiliarity and concerns about usability. Similarly Kuo et al. (2009) observed that older adults view robots as less intuitive and more impersonal than younger users. This study extends such findings by demonstrating that baseline attitudes are not immutable and can be mitigated through targeted interventions.

The findings also complement studies that explore how technology influences acceptance (Daruwala, 2024). Noted that younger adults’ enthusiasm for robots often stems from their association with innovation and progress, while older adults may perceive robots as threatening or alienating. This study confirms this dichotomy but demonstrates that well-crafted interventions, such as empathetic gestures and narratives, can successfully shift perceptions across generational divides.

### 12.1 Age-specific attitudes and emotional engagement

Age significantly influenced attitudes toward humanlike robots, particularly in emotional engagement and perceptions of physical interaction. Older adults in this study reported greater discomfort with robots, likely due to limited familiarity with and confidence in emerging technologies (Lee and Yen, 2023; Kuo et al., 2009). In contrast, younger adults displayed higher enthusiasm for technological advancements, aligning with broader trends of greater acceptance among younger age groups (Daruwala, 2024). These generational differences emphasise the need to tailor communication strategies and designs to meet the unique needs of different age groups.

Including older participants in the experimental group confirms that the intervention effectively improved emotional engagement across all age groups, even among populations with higher initial suspicion toward robots. However, a statistically significant negative correlation between age and perceived interpersonal connection (r = −0.202, p = 0.002) and positive affect (r = −0.140, p = 0.035) indicates that older adults reported more minor improvements than younger participants, contrary to the hypothesis (H3b). This finding underscores the importance of age-specific adjustments in robot design and intervention strategies. Incorporating additional sensory cues, extended exposure, or culturally familiar narratives may help older participants develop stronger emotional connections with robots.

## 13 Practical media and marketing relevance

Visual and textual interventions are adaptable and scalable, making them effective tools for promoting robots across various industries. Marketing campaigns that portray service robots performing familiar and relatable tasks have been shown to build trust and shape positive public perceptions (Qian and Wan, 2024; Iwamoto et al., 2022; Law et al., 2021). Additionally, service robots themselves can act as promotional tools. Incorporating multimodal representations, including images and videos of human-like service robots, businesses can attract customer attention, optimise promotional efforts, and enhance customer loyalty. Research has indicated that these strategies can result in notable sales growth, with increases ranging from 20% to several-fold (Kapustina et al., 2019).

The findings of this study highlight the potential of vivid visual and textual representations to change attitudes and improve perceptions of robots. The interventions used in this research, depicting service robots with humanlike gestures and relatable narratives, effectively reduced negative attitudes, increased positive affect, and improved perceived interpersonal connection to robots. These findings are supported by earlier research showing that visual imagery and concrete narratives enhance mental visualisation and encourage favourable attitudes (Babin and Burns, 1997). Demonstrated that visual imagery strengthens perceptions of advertisements and brands, while (Dasgupta and Greenwald, 2001) showed that positive visual exemplars can influence implicit attitudes even when explicit evaluations remain unaffected.

Given that the intervention produced measurable improvements in attitudes, emotional responses, and interpersonal connection, this study adds to existing research suggesting that human-like representations of service robots may facilitate consumer acceptance in service contexts. In sectors such as hospitality and caregiving, where emotional engagement is critical, humanlike features could improve perceptions of warmth, trust, and liveliness. Marketing strategies that highlight humanlike gestures and expressions, make robots more relatable and increase user acceptance. Evidence from the Henna Hotel in Japan, recognised as the first robot-operated hotel, indicates that the use of human-like imagery enhances consumer attitudes toward robotic technologies (Yu, 2020). Additionally, humanlike gestures activate shared cognitive schemas (Epley et al., 2007), promoting familiarity and encouraging emotional engagement (Orefice et al., 2016).

These insights have practical implications for anxious (Daruwala, 2024) or hesitant stakeholders and decision-makers considering the adoption of robotic technologies. Resistance to such technologies often stems from societal misgivings and perceived barriers (Castelo et al., 2019; Mende et al., 2019). This study shows that positive visual and textual portrayals can build trust and acceptance among users and stakeholders, reinforcing the notion that cultivating empathic interactions between humans and robots can support the development of a more compassionate and socially attuned technological future (Paiva et al., 2017).

To address these challenges, marketing strategies should raise awareness, encourage trial usage, and demonstrate service robots’ functional and emotional benefits (Noble and Mende, 2023). Visual and textual interventions, as utilised in this study, are effective tools for countering hesitancy and showcasing the value that robots can bring. Presenting relatable scenarios and humanlike behaviours provides a practical way to overcome resistance and promote the adoption of robotic technologies across diverse contexts.

## 14 Limitations and future directions

This study highlights the potential of visual and textual interventions to reduce negative attitudes and enhance emotional engagement with humanlike robots. However, limitations and opportunities for further research merit consideration.

A potential limitation of this study concerns the differential time spent in the experimental versus control conditions. Specifically, the experimental group was exposed to a short video (approximately 30 s), a narrative vignette (approximately 1 minute), and accompanying imagery, which amounted to an additional 2 minutes of engagement. While this temporal discrepancy is minimal, the added content could contribute to a mere exposure effect, whereby increased familiarity alone, rather than the nature of the content, may influence participants’ attitudes (Fiolka et al., 2024).

However, given the brevity of the additional material and the intervention’s theoretical grounding in embodied cognition and affective priming, it is reasonable to infer that the content, not duration alone, drove the observed changes in attitudes and perceived interpersonal connection. Nonetheless, future studies could control for exposure time by including neutral stimuli of equivalent duration in the control condition allowing for a more precise examination of the specific effects of multimodal human-like cues.

Cultural norms significantly shape how cues, such as gestures, are interpreted. Gestures like a handshake or bow may evoke varying emotional responses across cultures (Katsumi et al., 2017). Future research should explore culturally tailored interventions to ensure robot designs and communication strategies resonate with diverse global populations.

While this study focused on visual and textual cues to assess non-physical interactions, physical presence plays a pivotal role in human perceptions of service robots (Li, 2015). As (Broadbent, 2017) (2017) notes, individuals tend to experience stronger social and emotional resonance with physically embodied robots than virtual agents or computers, highlighting the importance of tangible presence in shaping relational dynamics with artificial entities. Moreover, incorporating additional sensory modalities such as haptic feedback, voice modulation, or auditory cues could enhance emotional engagement (Prasad et al., 2022). Exploring how these multisensory elements amplify robots’ perceived closeness and relatability represents a critical next step in human-robot interaction research.

The durability of intervention effects remains an open question. Longitudinal studies are needed to determine whether initial positive changes in attitudes and emotional engagement persist over repeated interactions. Understanding this evolution is crucial for designing robots that maintain long-term appeal and relevance.

Service Robots serve varied roles across industries, from healthcare to retail. Future research should evaluate how interventions and visual portrayals perform in specific contexts. Caregiving robots might prioritise empathetic gestures and narratives (Robinson and Nejat, 2022), while retail robots could focus on task efficiency and functionality (Vinoi et al., 2025).

This study identified age and gender-related differences in attitudes toward robots, underscoring the need for inclusive, user-centred designs. Testing gender and age-sensitive robots in the real world can inform strategies for addressing diverse user expectations.

Addressing these areas will enable future research to refine strategies for designing inclusive and adaptable robots. Such advancements will deepen our understanding of human-robot interaction, enabling robots to build meaningful and sustainable relationships in varied social, cultural, and professional contexts.

## 15 Conclusion

The current findings demonstrate the significant potential of visual and textual interventions in shaping emotional engagement with humanlike service robots. These strategies offer a practical framework for enhancing human-robot interaction (HRI) by reducing negative attitudes, increasing positive affect, and nurturing perceived interpersonal connection. Through carefully constructed imagery, video, and narrative, the research highlights the value of early-stage communication in influencing perceptions of humanlike robots.

This study extends prior empathy models by demonstrating that short-form multimodal interventions (e.g., imagery and vignettes) can meaningfully alter affective attitudes in non-physical HRI contexts. Empathetic narratives and relatable gestures, such as a handshake, help humanise robots, increase relatability, and build stronger consumer connections.

As humanlike service robots become more prevalent in service industries, these findings offer practical guidance for designing visual and narrative materials that present robots as approachable, functional, and emotionally engaging. By emphasising both functional utility and emotional value, positive portrayals in advertising can promote trust, reduce scepticism, and support broader public acceptance.

Furthermore, this research underscores the importance of tailoring interventions to demographic variables such as age and gender, enhancing their relevance and emotional resonance. These insights are especially valuable in applications where emotional connection is critical to user satisfaction and trust.

Ultimately, this study contributes to the broader psychological understanding of how humans perceive humanlike service robots and offers actionable insights for marketing, design, and public communication strategies. It lays a foundation for promoting meaningful human–robot relationships and successful societal integration of robotic technologies.

## Data Availability

The raw data supporting the conclusions of this article will be made available by the authors, without undue reservation.

## Appendix A

At a quaint local café, Aria strikes up an unexpected conversation with a humanoid robot named Atlas. Initially guarded, she gradually warms to the service robot’s presence as their dialogue unfolds, covering various topics. During their exchange, Atlas places a gentle hand on her shoulder and offers a handshake. The startlingly human texture of Atlas’s synthetic skin surprises and reassures her, momentarily easing her apprehension. However, her unease returns when the discussion shifts to the concept of robot emotions. This sparks a cascade of thoughts for Aria as she begins to reflect on the nature of touch, emotional authenticity, and the broader societal implications of human-robot interactions. The encounter ultimately challenges her preconceived notions, igniting a curiosity about the evolving relationship between humans and robots and what it might mean for the future.
